# Detecting DNA: An Overview of DNA Recognition by Inflammasomes and Protection against Bacterial Respiratory Infections

**DOI:** 10.3390/cells11101681

**Published:** 2022-05-19

**Authors:** Juselyn D. Tupik, Justin W. Markov Madanick, Hannah M. Ivester, Irving C. Allen

**Affiliations:** 1 Department of Biomedical Sciences and Pathobiology, Virginia-Maryland College of Veterinary Medicine, Virginia Tech, Blacksburg, VA 24061, USA; jdtupik@vt.edu (J.D.T.); justinm99@vt.edu (J.W.M.M.); hivester@vt.edu (H.M.I.); 2 Department of Basic Science Education, Virginia Tech Carilion School of Medicine, Roanoke, VA 24016, USA

**Keywords:** inflammasome, NLRP3, AIM2, IFI16, DNA, STING, ZBP1, Type I IFN, bacterial respiratory infection, pyroptosis

## Abstract

The innate immune system plays a key role in modulating host immune defense during bacterial disease. Upon sensing pathogen-associated molecular patterns (PAMPs), the multi-protein complex known as the inflammasome serves a protective role against bacteria burden through facilitating pathogen clearance and bacteria lysis. This can occur through two mechanisms: (1) the cleavage of pro-inflammatory cytokines IL-1β/IL-18 and (2) the initiation of inflammatory cell death termed pyroptosis. In recent literature, AIM2-like Receptor (ALR) and Nod-like Receptor (NLR) inflammasome activation has been implicated in host protection following recognition of bacterial DNA. Here, we review current literature synthesizing mechanisms of DNA recognition by inflammasomes during bacterial respiratory disease. This process can occur through direct sensing of DNA or indirectly by sensing pathogen-associated intracellular changes. Additionally, DNA recognition may be assisted through inflammasome–inflammasome interactions, specifically non-canonical inflammasome activation of NLRP3, and crosstalk with the interferon-inducible DNA sensors Stimulator of Interferon Genes (STING) and Z-DNA Binding Protein-1 (ZBP1). Ultimately, bacterial DNA sensing by inflammasomes is highly protective during respiratory disease, emphasizing the importance of inflammasome involvement in the respiratory tract.

## 1. Introduction

### 1.1. Inflammasome Sensing of Bacteria

**Figure 1:**
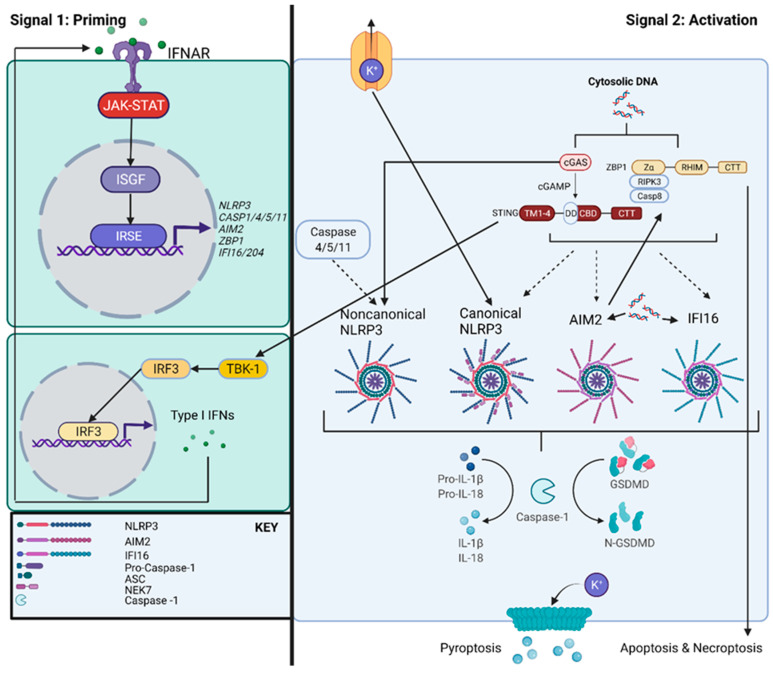
Mechanism of DNA effectors STING, ZBP1, and inflammasome activation in response to DNA. Inflammasome initiation of inflammatory cytokine activation and pyroptosis following nucleic acid stimulation can occur directly in the ALR inflammasomes AIM2 and IFI16. However, inflammasome activation to DNA, particularly by the NLR inflammasome NLRP3, may also be regulated by caspase-11/4/5 activation or the DNA sensing effectors STING and ZBP1. Upon sensing of DNA, effector STING in the cGAS axis induces the production of Type I IFNs, initiating IFN-alpha/beta receptor (IFNAR) and JAK-STAT activation of Interferon-stimulated Response Elements (IRSEs). This, in turn, promotes priming of inflammasome components and DNA effectors. The DNA sensing effector ZBP1 also initiates inflammasome activation and multiple forms of cell death in the proposed PAN-optosome pathway of cell death [3,4,5,6,7,8,9,10,11,12]. Created with BioRender.com (Agreement #AG23XLKR9E; accessed on 16 May 2022).

The innate immune system serves as one of the primary mechanisms of host–pathogen defense. One key effector in this system is a multi-protein complex termed the inflammasome (Figure 1) [1]. The canonical inflammasome typically consists of three components: (1) a Pattern Recognition Receptor (PRR) that senses pathogen components, (2) the adaptor protein Apoptosis-associated Speck-like protein containing a CARD (ASC), and (3) activated caspase-1 that cleaves and activates cytokines and other proteins. Inflammasomes first assemble in response to PRR sensing of conserved genetic motifs known as pathogen or damage-associated molecular patterns (PAMPS/DAMPS) [1]. Inflammasomes sense LPS, bacterial surface proteins, and nucleic acids, which facilitates activation and host defense [2]. Inflammasome-forming PRRs consist of the intracellular NOD-like receptors (NLRs) and AIM2-like receptors (ALRs) (Figure 1).

In particular, inflammasome-forming NLRs are characterized by three primary domains. First, NLRs contain a Leucine-rich Repeat, or LRR, domain that directly senses PAMPs and DAMPs [1]. They also contain a Nucleotide Binding and Oligomerization, or NACHT, domain that is involved in NLR oligomerization and scaffolding following PAMP/DAMP recognition [1]. Finally, NLRs have variable N-terminal domains, typically including a pyrin domain or caspase-activating recruitment domain (CARD) that facilitate multi-protein interactions to form the inflammasome [1]. In contrast, the inflammasome-forming ALRs do not contain an LRR or oligomerization domain. Instead, they have hematopoietic interferon-inducible nuclear proteins with a 200-amino acid repeat, or HIN-200, domain that senses nucleic acids directly [13]. Inflammasome-forming ALRs also contain a pyrin domain to facilitate inflammasome formation. Upon PRR recognition of PAMPs/DAMPs, NLRs bind to the adaptor protein ASC through pyrin domain or CARD interactions and recruit pro-caspases, such as pro-caspase-1, to form the core inflammasome unit [14]. This multiprotein complex cleaves pro-caspase into its mature form, which is then free to cleave target proteins [1,14]. 

Inflammasome activation initiates two primary cellular responses: (1) cleavage and maturation of the pro-inflammatory cytokines IL-1β and IL-18 and (2) initiation of inflammatory cell death termed pyroptosis [1]. In canonical inflammasome activation, pyroptosis occurs through caspase-1-mediated cleavage of the N-terminus of the protein gasdermin-D (GSDMD), which can initiate pore formation and an ion imbalance in the cell leading to cell lysis [15,16,17]. In addition to pore formation, GSDMD also contributes to a variety of antibacterial responses [18], including direct binding to bacteria to facilitate pathogen clearance [19], modulation of chemokines [20], and formation of neutrophil extracellular traps [21,22]. In response to bacterial infection, the inflammasome cleavage of pro-inflammatory cytokines and initiation of pyroptosis is highly activated in response to a variety of PAMPs/DAMPs [23]. Specifically, pyroptosis is highly dependent on ASC speck formation to form the pyroptosome [24]. This dependence may result from scaffolding formation by ASC, leading to enhanced recruitment of caspase-1 [24]. In the non-canonical inflammasome, caspase-11 CARD motifs in mice, or caspase-4 and 5 CARD motifs in humans, directly cleave the N-terminal domain of GSDMD resulting in pyroptosis [25,26]. The non-canonical inflammasome is also implicated in activation of the NLRP3 inflammasome through a mechanism that is currently not well-defined, leading to the secretion of pro-inflammatory cytokines (Figure 1) [27]. Ultimately, there are two signals involved in the regulation and activation of both canonical and non-canonical inflammasomes. Signal 1 involves inflammasome priming and promotes the transcriptional generation of inflammasome components and immature inflammatory cytokines. Signal 2 promotes inflammasome activation, cleavage of cytokines through post-translational processing, and initiation of pyroptosis (Figure 1) [1]. PAMP or DAMP signaling can mediate activation of either of these signals, making it essential to assess both signals in the context of bacterial infections.

### 1.2. Nucleic Acid Sensing by Inflammasomes

The most characterized inflammasome with regards to DNA sensing is the AIM2 inflammasome (Figure 1). AIM2, along with IFI16 (mouse homologue Ifi204), belong to the HIN-200 family [13,28]. Situated near the C-terminus, the HIN-200 domain facilitates the formation of the ALR inflammasome following recognition of cytoplasmic DNA [29]. DNA binding occurs through the HIN-200 domain’s two consecutive oligonucleotide/oligosaccharide-binding folds, or OB domains [29]. The negative charge of this region promotes binding with the highly positive sugar phosphate backbone of DNA [30]. This binding of DNA is also non-specific due to the HIN-200 family’s high conservation of amino acids, suggesting that DNA sensing by ALR inflammasomes is designed to recognize conserved DNA molecular patterns found in many organisms, including host DNA [29,30]. Within the ALR protein family, AIM2 contains only one HIN domain while IFI16 contains two, HIN-A and HIN-B [6,31]. IFI16 also contains a nuclear localization signal (NLS) and can therefore sense DNA in the nucleus in addition to the cytoplasm following inhibition of the NLS through post-translational acetylation [32]. Because ALR inflammasomes do not contain an oligomerization domain, such as the NACHT domain of NLRs, it is probable that dsDNA may also assist as the oligomerization platform for scaffold formation of the inflammasome [33]. For instance, the pyrin domains of the ALR inflammasomes can assist with DNA binding through filament formation on dsDNA [34]. Furthermore, DNA sensing may also occur in the N-terminal domain of some ALR inflammasomes [29]. Ultimately, these data suggest that the DNA sensing is highly regulated by inflammasome activation, with both ALR domains serving to facilitate dsDNA recognition.

Although the DNA sensing domain of the ALR inflammasomes has been well characterized, the role that NLR inflammasomes play in nucleic acid recognition and binding is less defined. Of the NLR inflammasomes, the best characterized in response to nucleic acid sensing has been the NLRP3 inflammasome. NLRP3 senses a diverse array of dissimilar molecular structures, including DAMPs such as ATP, K+ efflux, ROS species, and destabilization debris of lysosomes and phagosomes [35]. In response to pathogen invasion, the NLRP3 inflammasome senses viruses and bacteria, specifically inducing inflammasome formation and pyroptosis in response to pathogen nucleic acids [35]. Unlike the ALR inflammasomes, nucleic acid sensing by the NLRP3 inflammasome does not occur directly through the LRR domain and, instead, is instigated indirectly through relatively unknown cellular events, likely requiring upstream cytosolic DNA sensors for activation [36]. For instance, NLRP3 activation can occur in response to non-canonical inflammasome activation to bacterial PAMPs, which facilitates inflammasome crosstalk and activates the ATP-releasing channel pannexin-1, triggering ATP release and K+ efflux [36]. Furthermore, NLRP3 expression and activation may be activated by other DNA sensing effectors, either through regulation of inflammasome priming or stimulation of NLRP3 activation [37,38].

## 2. Bacterial DNA Sensing Effectors Associated with Inflammasome Activation

### 2.1. Stimulator of Interferon Genes (STING)

Stimulator of Interferon Genes (STING) is one of the main DNA sensors in response to pathogen infection. STING is a general sensor of nucleic acids, including self-DNA, DNA and RNA viruses, and both Gram-positive and Gram-negative bacteria, suggesting versatility in pathogen recognition [39]. STING consists of an N-terminal transmembrane domain that anchors to the endoplasmic reticulum (ER), a cytoplasmic heterodimer consisting of a Ligand Binding Domain (LBD) as a part of the C-terminal Domain (CTD), and relatively uncharacterized C-terminal tail (CTT) involved in phosphorylation and interferon (IFN) binding (Figure 1) [3,4]. As a predominant adaptor protein in DNA recognition, STING interacts with the DNA sensor cyclic GMP-AMP synthase (cGAS). cGAS is a nucleotidyl transferase enzyme which directly binds DNA, preferably dsDNA longer than 45 base pairs [39]. Once activated through DNA binding, cGAS converts GTP and ATP into the second messenger cyclic GMP-AMP (cGAMP). The production of cGAMP is further enhanced by the formation of liquid droplets that function to increase cGAS concentration [39]. After this process, cGAMP binds to STING, promoting STING oligomerization and release from the ER [39]. STING also directly binds to primary sensors of DNA, including multiple DExD/H-box helicases (DDX) proteins [3]. STING regulates NLR, ALR, and non-canonical inflammasome activation through priming of inflammasome molecular components (Figure 1). This occurs primarily through STING interactions with Tank Binding Kinase 1 (TBK1), which phosphorylates STING and allows for Interferon Regulatory Factor 3 (IRF3) activation and translocation into the nucleus to stimulate transcription of Type I IFNs [39]. Once Type I IFNs are released extracellularly, they can bind to Interferon Associated Receptor (IFNAR), which is involved in the JAK-STAT pathway and induces transcription of caspase-1 (canonical), caspase-4/5 and 11 (non-canonical), AIM2 (ALR), and NLRP3 (NLR) (Figure 1) [5].

STING can regulate inflammasome activation through multiple unique pathways depending on specific inflammasomes. With regards to the NLRP3 inflammasome, STING initiates NLRP3 recruitment to the ER to bind to the NACHT and LRR domains with one of its transmembrane domains (Figure 1) [38]. These STING-NLRP3 interactions increase the deubiquitination of NLRP3 to enhance NLRP3 inflammasome assembly and secretion of IL-1β [38]. Furthermore, STING-NLRP3 facilitates the oligomerization of ASC, possibly promoting formation of the pyroptosome. In a separate mechanism, STING-mediated lytic cell death also instigates the production of a K+ efflux. This, in turn, triggers NLRP3 activation and subsequent pyroptosis [40]. STING can also initiate cell death through direct binding with inflammasome components. Upon nucleic acid sensing, IFI16 complexes with STING through binding to its HIN domains to initiate cell death and regulate apoptotic genes and IFN signaling (Figure 1) [6,41,42]. This indicates that IFI16 activation in the cytosol can be highly regulated by STING in response to pathogenic DNA and may possibly influence inflammasome priming through IFN production. Although non-canonical inflammasome initiation of cell death is associated with bacterial infection, such as in *Chlamydia trachomatis* infection, STING has not been shown to directly interact with caspase-11 to mediate pyroptosis [43].

Inflammasome interactions with STING may also serve to regulate overzealous endogenous DNA recognition. Overexpression of STING has been implicated in autoimmune diseases such as Systemic Lupus Erythematosus (SLE) and Aicardi–Goutières syndrome [44]. One of the attributed causes of enhanced SLE is from increased IFN activity and release of apoptotic-derived membrane vesicles [45]. Therefore, it may be advantageous for the innate immune system to control cell death mechanisms in response to self-DNA. One mechanism in which regulation can occur is through degradation of IFI16 by STING. This process occurs through STING interactions with the N-terminal region of IFI16, leading to IFI16 degradation through the ubiquitin–proteasome pathway [42,46]. Ultimately, IFI16 degradation decreases cell death and IFN production [42,46]. These data suggest that STING serves as a negative feedback loop against autoimmune disease. However, in return, the AIM2 inflammasome may also serve as a checkpoint for overzealous STING activation. In murine studies, AIM2 deficiency was found to increase Type I IFN expression and activation of STING [47]. This was augmented through the cGAS axis, with cGAMP elevated in *Asc^−/−^* cells [47]. Although STING may serve a protective function during bacterial or viral infection, there are mechanisms in place to regulate self-DNA and overzealous inflammasome activation.

### 2.2. Z-DNA Binding Protein 1 (ZBP1)

ZBP1, or z-DNA Binding Protein 1 (formerly known as DAI and DLM1), is another interferon-induced sensor involved in the cytosolic recognition of nucleic acids. As an IFN-inducible gene, ZBP1 expression and activation can be controlled through pathogen induction of IFNs, specifically IRF1 [48]. However, ZBP1 activation also occurs through direct sensing of nucleic acid ligands. ZBP1 senses dsDNA through its N terminal Z-DNA binding domain (ZBD) (Figure 1) [7]. Although most literature characterizes ZBP1 as a sensor of viral nucleic acids, particularly in response to influenza virus, recent studies on fungal pathogens *Candida albicans* and *Aspergillus fumigatus* [49] and the bacterium *Yersinia pseudotuberculosis* [50] suggests the wide diversity of pathogens sensed by ZBP1 and warrants further investigation of ZBP1 ligands. ZBP1 also contains two other molecular regions, a RIP homotypic interaction motif (RHIM) domain that regulates programmed cell death and interferon (IFN) production, and an undefined and conserved C-terminal domain that promotes signal transduction predominantly of IFNs (Figure 1) [7,8]. The RHIM domain recruits Receptor Interacting Serine/Threonine Kinases (RIPK), which through their death domain and recruitment of caspase-8 lead to the induction of apoptosis, necroptosis, and pyroptosis [7]. These forms of programmed cell death are thought to work together under regulation by central molecular hubs to make up the hypothesized master pathway of coordinated cell death termed PAN-optosis [9,37]. ZBP1 is suggested to serve as a central molecular mediator of PAN-optosis, implying that DNA recognition is critical for initiating host cell death.

Inflammasome initiation of pyroptotic cell death is highly linked with DNA sensing by ZBP1 (Figure 1). Specifically, ZBP1 interactions with NLRP3 and AIM2 inflammasomes are described in the context of the bacteria and viruses such as *Francisella novicida* and influenza virus, indicating the importance of ZBP1 in host–pathogen interactions during respiratory infection [51,52,53]. During pathogen infection, ZBP1 can be essential for activation of NLRP3, possibly recruiting NLRP3 after polyubiquitination during infection [54]. Subsequently, ZBP1 promotes release of IL-1β/IL-18 and the initiation of cell death, including pyroptosis, apoptosis, and necroptosis, further supporting the PAN-optosome hypothesis [51,55]. In AIM2 inflammasome activation during *F. novicida* infection, ZBP1 and pyrin expression have been implicated in forming the AIM2 PAN-optosome in complex with ASC to promote cell death [52]. The AIM2 inflammasome is also suggested to regulate ZBP1 and pyrin expression through (1) the initiation of IFN signaling and (2) inhibition of Rho-GTP activity to activate pyrin [52] (Figure 1). Although the AIM2 PAN-optosome is relatively understudied in bacterial infection, this work suggests that other bacteria that perform these two functions may also trigger the initiation of cell death through similar mechanisms. ZBP1 and the IFI16 have also been associated during pathogen infection [53]. After herpes simplex virus stimulation, ZBP1 and IFI16 expression is upregulated and promotes secretion of IFNα/β with DNA colocalization to both proteins [53,56]. This is hypothesized to be driven by ZBP1 DNA recognition, potentially impacting IFI16 activation in the nucleus downstream. These data suggest that ZBP1 interactions with multiple inflammasomes and initiation of cell death may depend on a variety of other pathways such as IFN signaling evoked by specific PAMPs.

## 3. Bacterial Respiratory Diseases Implicated with DNA-Induced Inflammasome Activation

Many bacterial diseases with defined mechanisms of DNA sensing are associated with bacteria-induced inflammasome activation (Table 1). These diseases consist of a variety of Gram-negative and Gram-positive bacteria, with many unrelated protein structures, virulence factors, and tactics for avoiding host defenses. Despite these differences, DNA sensing occurs through similar mechanisms, including IFN regulation of inflammasome components through the cGAS–STING axis, inflammasome initiation of cytokine secretion and pyroptosis, and ZBP1 regulation of cell death (Table 1).

### 3.1. Brucella *spp.*

*Brucella* spp., such as *B. abortus*, *B. melitensis*, *B. suis*, and *B. canis*, are the causative agents of the zoonotic disease brucellosis. *Brucella* are Gram-negative bacteria that reside in *Brucella*-containing vacuoles (BCVs) predominately in macrophages [57]. Upon infection, brucellosis can cause undulant fevers, joint pain, and a pulmonary response following inhalation [58]. During respiratory infections, the NLRP3 and AIM2 inflammasomes and caspase-11 protect against *Brucella abortus* through decreasing CFUs in the lungs and bronchoalveolar lavage fluid (BALF) [58]. In response to *Brucella* DNA, STING and the AIM2 inflammasome directly instigate caspase-1 activation and IL-1β secretion in macrophages in a cGAS-independent manner [59]. This is potentially aided by small interferon-inducible GTPases called guanylate-binding proteins (GBPs), which serve to promote bacteria release from BCVs and expose bacterial DNA [59]. GBPs may also promote caspase-11 induced pyroptosis, further controlling brucellosis [60]. This process can be regulated through IFN production, in which STING regulates GBP expression and subsequent protection against *Brucella* infection in vivo [59].

The AIM2 and NLRP3 inflammasomes are critical for caspase-1 activation and IL-1β secretion in macrophages and dendritic cells, which confer host protection during brucellosis [57,61]. ASC-dependent inflammasomes, particularly AIM2, may promote *Brucella* DNA recognition in vitro in macrophages, in which *Brucella* genomic DNA challenge in macrophages promotes IL-1β secretion and pyroptosis [58,62]. Interestingly, the protective function of the inflammasome against *Brucella* DNA may result primarily from the initiation of pyroptosis [62,63]. In macrophages, ASC-dependent inflammasomes induce pyroptosis in a GSDMD-dependent manner [62]. This mechanism is protective against brucellosis in murine models, particularly through caspase-1/11 restricting *Brucella*-induced joint swelling and bacterial load in the spleen [60,63]. Although this mechanism has not yet been described in the lungs, it is possible that pyroptosis may also drive the protective function of the inflammasome following sensing during *Brucella* pulmonary infection.

### 3.2. Burkholderia *spp.*

The Gram-negative bacterium *Burkholderia*, specifically *B. pseudomallei*, causes the tropical disease melioidosis, a severe respiratory disease that causes pneumonia-like symptoms [64]. *Burkholderia* virulence can be derived from its ability to escape the phagosome and replicate intracellularly [64]. Despite this tactic for host immune evasion, the NLRP3, NLRC4, and caspase-11 inflammasomes are highly involved in decreasing bacterial load in the lungs. During infection, NLRP3 is the primary producer of IL-1β and IL-18 [64,65]. Although IL-18 mediates a protective role against *Burkholderia* through inducing IFNγ production, IL-1β induces recruitment of neutrophils and promotes lung tissue damage [64,65]. The most protective function of the inflammasome during melioidosis is NLRC4 and caspase-11-induced pyroptosis [26,64,66,67,68]. Current literature suggests that NLRC4 and caspase-11 are activated at different timelines during melioidosis, in which NLRC4 activates pyroptosis in early phases of infection and induces IFNγ, reducing intracellular bacterial load and possibly primes caspase-11 [64,68]. In late-stage infections, caspase-11 plays a predominate role in initiating pyroptosis through cleavage of GSDMD in addition to NLRP3 activation [26,67]. This has been demonstrated using lung epithelial cells, which lack inflammasome components except for caspase-11 [67]. Ultimately, pyroptosis and other forms of cell death are highly effective mechanisms of bacteria clearance [69]. In fact, the PAN-optosis master pathway of cell death may protect against host mortality through limiting *Burkholderia thailandensis* cell-to-cell fusion and replication [70].

Although there are clear mechanisms of inflammasome activation during *Burkholderia* infection, there are currently no established pathways of inflammasome recognition of *Burkholderia* DNA. However, DNA recognition by STING could play a currently undefined role in inflammasome priming or cell death pathways. For instance, cGAS-STING induces IFN transcription in response to *Burkholderia* infection [71]. IFN production also induces GBP-mediated activation of caspase-11, which induces pyroptosis [72]. These studies suggest that DNA sensing could promote inflammasome activation, possibly through IFN-mediated pathways, including inflammasome priming. Another inflammasome pathway activated in response to *Burkholderia* is pyrin inflammasomes. In human THP-1 cells, pyrin inflammasomes activate the canonical inflammasome during *Burkholderia cenocepacia* infection to promote IL-1β secretion and cell death [73]. Interestingly, the AIM2 inflammasome, while structurally similar to pyrin inflammasomes, is understudied during *Burkholderia* infection, warranting further investigation into mechanisms of ALR inflammasome sensing.

### 3.3. Francisella tularensis/novicida

*Francisella tularensis* is a Gram-negative, facultative intracellular bacterium and the causative agent of tularemia. Tularemia often leads to flu-like symptoms, including fevers and swollen lymph nodes. In severe cases, tularemia can lead to life-threatening respiratory disease upon inhalation [74]. *Francisella* spp. are implicated in inflammasome recognition by both NLR and ALR inflammasomes. However, the NLRP3 inflammasome plays different roles in host defense in mice and humans. In humans, the NLRP3 inflammasome assembly is triggered by *Francisella* in an undescribed mechanism and induces IL-1β expression to protect against *Francisella* infection [75]. In contrast, in vivo and in vitro experiments using *Nlrp3*^−/−^ murine models exhibited similar IL-1β and IL-18 expression to wildtype mice. Furthermore, *Nlrp3*-deficient mice exhibit reduced lung pathology, possibly due to NLRP3-induced cell death contributing to the negative pathology during tularemia [76]. These data suggest that the role of NLRP3 in mice may not serve a protective function, emphasizing the role of other inflammasomes during *Francisella* infection.

Beyond the NLRP3 inflammasome, the AIM2 inflammasome is most described in response to *Francisella*. AIM2 senses DNA from *Francisella*, which binds with the HIN-200 domain of AIM2 and leads to ASC speck recruitment to form the pyroptosome [77,78,79]. Within phagocytic immune cells, *Francisella* initiates pyroptosis in an AIM2 and ASC-dependent manner that is independent of caspase-1, although IL-1β relies on caspase-1 recruitment [77,78,79]. Studies also show that GSDMD also plays an essential role in initiating pyroptosis to *Francisella novicida* [80]. This effect of cell death is critical in protecting against bacteria burden and mortality in mouse models [78]. AIM2-mediated cell death can also be aided through DNA effectors. For instance, ZBP1 is implicated in formation of the AIM2 PAN-optosome during *Francisella* infection [52], suggesting the importance of upstream signaling pathways in DNA-mediated cell death.

AIM2 inflammasome activation may also be regulated by IFN signaling. During *Francisella* infection, IFN signaling through IFNAR in a cGAS-STING-IRF-dependent manner can increase AIM2 priming [79,81]. Recent literature suggests that IRF1/3 is required for AIM2 activation and subsequent release of IL-1β and IL-18 and AIM2-dependent cell death [79,82]. Furthermore, IRF1 also induces production of GBPs, which serve to improve caspase-1 activation, in addition to IL-1β and IL-18 release and cell death by assisting with *Francisella* release from vacuoles [81,83]. Ifi204, the murine homologue of IFI16, is also implicated with STING-dependent signaling in vitro [84]. Although postulated, the ability of ifi204 to form an inflammasome as in its human homologue IFI16 is not well-demonstrated. Regardless, these data highlight the interconnected nature of IFN signaling in inflammasome activation to DNA. Although AIM2 serves a protective role in the host against *Francisella,* IFN signaling alone is suggested to decrease host survival. This is possibly due to IFN signaling promoting the initiation of apoptosis [85]. In fact, the AIM2 inflammasome may be intertwined with apoptosis through caspase-8 complexing with AIM2/ASC specks [86]. Interestingly, GSDMD activation in pyroptosis during *Francisella* infection can decrease IFN signaling through a K+ efflux [87].

Because of the inflammasome’s importance in protecting against *Francisella*, some virulence factors have evolved to degrade inflammasome activation to subvert bacterial destruction. *Francisella* lack a traditional LPS structure, which could possibly subvert non-canonical inflammasome activation by caspase-11 [88]. To regulate canonical inflammasome activation, the *FTL_0325* gene decreases expression of pro-IL-1β and priming of inflammasomes [89]. The gene *mviN*, a lipid II flippase, also decreases activation of the AIM2 inflammasome [90]. These genes hinder bacteria and host cell lysis, which is essential for AIM2 recognition of *Francisella* DNA and implies that lysis is important for DNA recognition pathways [91].

### 3.4. Legionella pneumophila

*Legionella pneumophila*, the causative agent of Legionnaire’s disease, is an intracellular Gram-negative bacterium that leads to pneumonia, particularly in immunocompromised individuals [92]. *L. pneumophila* invade alveolar macrophages and other monocytes through *Legionella*-containing vacuoles (LCVs). However, upon emergence from LCVs, a variety of inflammasomes have been identified in recognition of *L. pneumophila.* The NAIP5/NLRC4 inflammasome is highly activated in response to *L. pneumophila*, particularly following recognition of flagellin [92,93]. NLRC4 in particular acts in a caspase-1, but ASC-independent mechanism to initiate release of IL-18 and pyroptosis in response to flagellin [93]. Similarly, NAIP5 promotes caspase-1 activation to initiate pyroptosis and restrict intracellular bacterial growth and apoptosis [94]. These studies could suggest that ASC involvement in the pyroptosome may be pathogen-specific, or predominantly involved in response to nucleic acid recognition.

In contrast to the NLRC4 inflammasome, the non-canonical inflammasome (caspase-11) is strongly linked with both ASC-dependent activation of caspase-1 and NLRP3 inflammasome involvement [95]. In vitro, caspase-11 induces NLRP3 and caspase-1 activation downstream of *L. pneumophila* recognition, promoting NLRC4-independent pyroptosis [95]. Interactions between inflammasomes may also be controlled by AIM2 recognition of *L. pneumophila* DNA. The AIM2 inflammasome, once activated, engages activation of caspase-1 and subsequent pore formation of the cell [96]. In complex with caspase-11 activation, damage to the cell membrane produces a K+ efflux, inducing NLRP3 activation [96]. Furthermore, involvement of caspase-11 may be regulated by IFN signaling in a STING-IRF3 dependent manner, as pyroptosis was severely deficient in macrophages deficient in IFNAR [95,97]. Expression of GBPs may also facilitate caspase-11 activation and pyroptosis following DNA recognition [98]. For instance, IFN production from GBPs occurs in a STING-dependent manner, promoting release of *L. pneumophila* DNA from LCVs and subsequent bacteria lysis [98,99]. In mice and humans that have impaired cGAS-STING signaling, there is higher bacterial load and frequency of severe Legionnaire’s disease [100]. Ultimately, these data indicate that IFN signaling within the GBP/cGAS–STING axis is protective against *L. pneumophila* in murine models through assisting with bacteria clearance [97].

To avoid host inflammasome defense, some *L. pneumophila* effectors are implicated in targeting inflammasome activation [101]. For instance, the SdhA effector, which makes up the Dot/Icm secretion system to block lysosomal fusion, prevents AIM2 activation [101]. Additionally, SdhA is hypothesized to enforce membrane integrity of the LCV, which prevents DNA from leaving the vacuole and subsequent AIM2 inflammasome activation. This *L. pneumophila* effector ultimately reinforces the positive role of inflammasome activation in promoting host defense.

### 3.5. Mycobacterium tuberculosis

*Mycobacterium tuberculosis* causes tuberculosis, one of the most prevalent bacterial respiratory infections worldwide, with an average of 1.6 million deaths per year [102]. One predominant pathological marker of tuberculosis is tissue necrosis in the lungs [102]. This can occur through various unregulated forms of cell death but is also exacerbated by pyroptosis following activation of the NLRP3 inflammasome [102,103]. In fact, the type VII secretion system and protein EST12 of *M. tuberculosis* are secreted by the bacterium to induce NLRP3-mediated pyroptosis. This occurs through methods such as membrane degradation inducing a K+ efflux or activation of NLRP3 through K48-linked deubiqutination [102,103]. Upon sensing DNA, the PAN-optosis pathway is also evoked by *M. tuberculosis* through upregulation of ZBP1 to establish a necroptotic environment in macrophages [104]. These data suggest that *M. tuberculosis* exploits inflammasome-mediated pyroptosis and cell death following DNA recognition to enhance bacteria dissemination in the lungs. However, one mechanism employed by the inflammasome to protect against intratracheal infection is NLRP3/ASC secretion of IL-1β and IL-18 [105]. This occurs in dendritic cells and protects against apoptosis [105]. Consequently, the *M. tuberculosis* phosphokinase PknF has evolved to avoid this protective inflammasome function by reducing NLRP3 activation, which deceases IL-1β and IL-18 secretion [106]. Ultimately, these studies highlight many pathways utilized to either promote harmful NLRP3 pyroptosis or limit NLRP3 cytokine secretion, subsequently promoting *M. tuberculosis* virulence.

AIM2 inflammasome activation following cytoplasmic DNA sensing is also protective against tuberculosis. Similar to NLRP3, AIM2 induction of IL-1β and IL-18 promote a protective Th1 response in the host [107]. In humans, single-nucleotide variant rs1103577 in *AIM2* elevates endogenous IL-1β, possibly protecting against pulmonary tuberculosis [108]. AIM2 may also protect against *M. tuberculosis* through AIM2-ASC interactions with STING, preventing STING interactions with TBK1 following DNA recognition and subsequent overactive IFN production [109,110]. Despite the negative implication of cGAS-STING activation during tuberculosis, some studies suggest the importance of antimicrobial activities by cGAS, such as cytokine production, autophagy, and dendritic cell activation in protection against infection [111,112,113]. These data suggest the importance of AIM2 regulation of the cGAS–STING axis and IFN production following DNA sensing. Consequently, to subvert AIM2 activation, *M. tuberculosis* uses its type VII secretion system as in the NLRP3 inflammasome to suppress AIM2 [114]. Ultimately, inflammasome sensing of DNA during tuberculosis is highly intertwined between the host and pathogen, balancing host cytokine defense with pathogen exploitation of pyroptosis and IFN production.

### 3.6. Staphylococcus aureus

*Staphylococcus aureus* is a Gram-positive bacterium and one of the major worldwide causes of bacteremia and, in severe cases, pneumonia [115]. The NLRP3 inflammasome is highly activated in response to *S. aureus* and plays conflicting roles with regard to infection. To protect against infection, NLRP3 and ASC are crucial for early clearance of bacteria [116]. In some cases, early initiation of necroptosis in combination with NLRP3 activation also protects against bacterial load and limits excessive inflammation [117]. However, the sustained inflammatory secretion of IL-1β/IL-18 by NLRP3 during *S. aureus* infection often leads to a harmful pro-inflammatory environment in the lungs, causing an over-influx of neutrophils and damaging tissue necrosis [118,119,120,121,122]. NLRP3-induced pyroptosis through K+ efflux in macrophages and epithelial cells causes sustained tissue damage in addition to neutrophil recruitment [118,119]. NLRP3 activation is further facilitated through the Staphylococcal virulence factor α-hemolysin and toxin secretion, which causes severe *S. aureus* pneumonia [120,121,122]. These data indicate that the NLRP3 inflammasome plays a predominately harmful role in *S. aureus* infection. However, *S. aureus* DNA sensing by STING and other interferon inducible families of proteins may serve to attenuate inflammasome activation. The STING/TBK1/IRF3 pathway produces IFN-β in response to *S. aureus* DNA, promoting bacterial autolysis to protect against infection [123]. Additionally, STING can protect against lung necroptosis to rescue host defense [124]. STING-mediated IFN production may also polarize macrophages towards an anti-inflammatory response, which, while advantageous for bacteria persistence, could also potentially decrease the pro-inflammatory environment induced in long-term infections by NLRP3 [125]. Furthermore, Ifi204 is associated with protection against *S. aureus* infection following DNA recognition, promoting extracellular bacterial killing and IFN production [126]. Although a direct mechanism of DNA recognition to regulation of the NLRP3 inflammasome has yet to be defined, it is possible that host sensing of DNA may be protective against tissue damage exploited by *S. aureus* inflammasome activation.

### 3.7. Streptococcus pneumoniae

*Streptococcus pneumoniae* is a Gram-positive bacterium that leads to the development of flu-like symptoms and colonization of the respiratory tract in pneumococcal disease [127]. In response to *S. pneumoniae*, ASC-dependent inflammasomes are critical for initiating a protective immune response against pneumococcal infection [128]. ASC is implicated in both AIM2 and NLRP3 inflammasome activation to *S. pneumoniae.* AIM2, in complex with ASC oligomerization, is essential for caspase-1 activation following direct Streptococcal DNA sensing [128]. This activation of the AIM2 inflammasome is protective against intranasal infection in murine models [129]. Although a direct DNA to NLRP3 activation mechanism has not been defined, NLRP3 stimulates IL-1β production in response to high doses of *S. pneumoniae* [130]. During *S. pneumoniae* infection, NLRP3 promotes inflammasome activation and pyroptosis in a variety of cell types. NLRP3 in microglial cells during pneumococcal meningitis and in corneal infection is shown to activate caspase-1 leading to IL-1β and IL-18 cleavage [131,132]. Additionally, NLRP3 in neutrophils is critical for IL-1β secretion [133]. However, in a transcriptomic analysis between gene expression of *Nlrp3^−/−^* and *Asc^−/−^* mice, genes regulating the adaptive immune response were significantly downregulated in *Asc^−/−^* mice compared to *Nlrp3^−/−^* mice [134]. This suggests that ASC may play a greater role in inflammasome activation against Streptococcal infection.

One of the main pathways involved in inflammasome regulation during pneumococcal disease is Type I IFN signaling. Specifically, AIM2 inflammasome activation during *S. pneumoniae* infection is regulated by Type I IFN production. In fact, IL-18 production is dependent on IFN signaling [135]. Intriguingly, NLRP3 is not dependent on IFN expression, emphasizing the importance of IFNs in AIM2 inflammasome activation instead [135]. IFN signaling is also influenced through DNA sensing effectors, in which the absence of STING and ZBP1 lead to a 50–100% decrease in *Ifnb* induction [136]. Specifically, IFN signaling can be mediated in a STING-dependent manner, initiating IRF3 signaling to produce Type I IFN in human and murine macrophages [137]. Ultimately, elevated levels of Type I IFN confer a protective effect in mice, with reduced bacteria counts in nasal lavage fluid and reduced lung inflammation [136]. As no defined mechanism of DNA activation has been shown for the NLRP3 inflammasome, it is possible that these effectors could promote NLRP3 activation and pyroptosis. For instance, RIPK3 initiates necroptosis in pneumococcal disease, triggering a ROS influx and subsequent activation of the NLRP3 inflammasome [138]. As ZBP1 complexes with RIPK3 in the PAN-optosome, it is possible that DNA sensing could influence NLRP3 activation through this undescribed mechanism.

## 4. Conclusions

As presented throughout, inflammasome sensing of bacterial respiratory diseases plays a critical and positive role in host defense. Of the seven bacteria discussed, DNA sensing and inflammasome activation are critical for cytokine secretion and cell death. Additionally, inflammasome activation is highly protective against bacterial burden and host mortality. More broadly, these data emphasize the protective role of pathogen DNA recognition by the innate immune system. Many of the bacteria included in this review have evolved highly sophisticated mechanisms of inflammasome immune evasion through means such as toxin secretion, reinforced vacuoles, and surface protein modifications, further illustrating the critical role that inflammasomes play in host defense. Despite these modifications, DNA recognition by inflammasomes is still able to recognize these pathogens and drive a vigorous host innate immune response.

In this review, we highlight the importance of pyroptosis following DNA recognition and its role in protection against bacterial infection. Of the respiratory diseases discussed here, we highlight the host initiation of pyroptosis through the non-canonical inflammasome, as well as the AIM2 and NLRP3 inflammasomes. These inflammasomes defend against respiratory infection through aiding in bacteria lysis and ultimately decrease pathogen load. Because of this protection, many bacterial toxins and virulence factors are designed to limit inflammasome activation or DNA sensing effectors. Furthermore, some bacteria can also exploit pyroptosis and cell death to instigate host tissue damage and necrosis. Ultimately, inflammasome initiation of pyroptosis following DNA recognition is an important, under-emphasized, and relatively undefined pathway in response to bacterial respiratory disease.

In recent literature, IFN signaling is suggested to promote inflammasome activation through enhancing inflammasome priming. Despite this emphasis, crosstalk between IFN-induced proteins and DNA sensors during inflammasome activation has not been highlighted. Here, we emphasize the integrated role of DNA sensing and IFN production with its impact on regulation of the inflammasome. For instance, through IFN signaling, production of interferon-inducible GBPs aid in bacteria and host cell lysis. Consequently, GBPs further enhance bacterial DNA release, which promotes DNA sensing by the ALR inflammasomes and production of host DAMPs for NLRP3 inflammasome activation. IFN signaling also influences the production of DNA sensing effectors during inflammasome activation. IFN-inducible DNA sensors STING and ZBP1 play critical roles for inducing inflammasome-medicated cytokine maturation and programmed cell death. Ultimately, the bacterial respiratory infections highlighted here are highly intertwined with IFN regulation of DNA sensing. Interestingly, in bacterial infections with inflammasome-induced pulmonary damage, IFN signaling is hypothesized to promote an anti-inflammatory environment or instigate antimicrobial activities. Therefore, IFN signaling may serve as a set of checks and balances on inflammasome activation during bacterial infections.

## Figures and Tables

**Table 1:** Mechanisms of inflammasome recognition of bacterial DNA associated with respiratory pathogens.

Bacteria	Mechanisms of DNA-Mediated Inflammasome Activation	Function in Disease	References
*Brucella* spp.	STING-dependent but cGAS independent recognition of DNA; DNA release is mediated by GBPs and regulates caspase-11 activation; DNA promotes activation of the AIM2/ASC inflammasome and induces IL-1β secretion and pyroptosis in dendritic cells and macrophages; some implications of NLRP3 activation but not in the induction of pyroptosis	Protective against bacterial burden; pulmonary protection in alveolar macrophages	[57,58,59,60,61,62,63]
*Burkholderia* spp.	cGAS–STING recognition triggering autophagic cell death; GBP induction of non-canonical inflammasome activation and IFN stimulation; Caspase-1 meditation of pyroptosis in macrophages; Caspase-11 inflammasome mediated cleavage of Gasdermin-D pyroptosis and NLRP3 activation; PAN-optosis initiation of pyroptosis and apoptosis	Protection against bacterial replication and spread in murine lungs and human epithelial cells through pyroptosis and cell death	[26,64,65,66,67,68,69,70,71,72,73]
*Francisella tularensis/novicida*	GBP/STING/Ifi204 initiation of IFN signaling; IFN regulation of AIM2 inflammasome components; AIM2/ASC initiation of cytokine release and pyroptosis/apoptosis; GSDMD regulation of IFN signaling	Protective against bacterial load in lungs and mortality in murine models	[52,74,75,76,77,78,79,80,81,82,83,84,85,86,87,88,89,90,91]
*Legionella pneumophila*	GBP/cGAS-STING initiation of IFN signaling and caspase-11 initiation of pyroptosis; AIM2/caspase-11 regulation of NLRP3 inflammasome activation	Protective against higher bacterial load and frequency of severe Legionnaire’s disease in humans	[92,93,94,95,96,97,98,99,100,101]
*Mycobacterium tuberculosis*	cGAS-STING activation of dendritic cells; ZBP1-mediated necroptosis; K+ efflux/ROS triggering NLRP3/ASC cytokine maturation and pyroptosis; IFN stimulation of AIM2 initiation of pyroptosis; AIM2 regulation of cGAS-STING overactivation of IFN signaling; caspase-11 initiation of pyroptosis	Protection against intratracheal infection through cytokine secretion; harmful when pyroptosis releases bacteria from phagosome	[102,103,104,105,106,107,108,109,110,111,112,113,114]
*Staphylococcus aureus*	STING-mediated suppression of necroptosis and IFN expression; Ifi204 initiation of IFN signaling and bacteria clearance; NLRP3/ASC activation with cytokine secretion and pyroptosis/necrosis; RIPK3 necroptosis initiation of bacteria clearance	Inflammasome protection against bacteria clearance but harmful initiation of excessive inflammation and mortality; DNA sensing may control excessive inflammation	[115,116,117,118,119,120,121,122,123,124,125,126]
*Streptococcus pneumoniae*	STING/ZBP1 mediation of IFN signaling and subsequent AIM2 component activation; AIM2/ASC and NLRP3 cytokine secretion and pyroptosis; RIPK3 initiation of NLRP3 activation and cell death	Reduced bacterial counts in nasal lavage fluid and reduced lung inflammation	[127,128,129,130,131,132,133,134,135,136,137,138]

## Data Availability

Not applicable.
